# Effect of Pelvic Floor Neurodynamics on Bladder Control and Quality of Life in Non-traumatic Post-operative Spinal Cord Injury

**DOI:** 10.7759/cureus.112839

**Published:** 2026-07-17

**Authors:** Sakshi P Kavitake, Suraj Kanase

**Affiliations:** 1 Department of Neurosciences, Krishna College of Physiotherapy, Krishna Vishwa Vidyapeeth (Deemed to be University), Karad, IND

**Keywords:** bladder dysfunction, neurogenic bladder, pelvic floor neurodynamics, physiotherapy rehabilitation, quality of life, spinal cord injury

## Abstract

Introduction

Spinal cord injury (SCI) results in profound neurological deficits, including bladder dysfunction that significantly impairs quality of life. Non-traumatic SCI, arising from spinal stenosis, tumours, or disc prolapse, accounts for a growing proportion of SCI cases globally. Neurogenic bladder dysfunction, manifesting as urgency, incontinence, or retention, remains one of the most debilitating sequelae. Pelvic floor neurodynamics (PFN), integrating neural mobilisation techniques with targeted pelvic floor rehabilitation, represents a novel therapeutic approach. However, its efficacy in the post-operative non-traumatic SCI population has not been formally evaluated.

Method

A randomised controlled trial was conducted at Krishna College of Physiotherapy in Karad, Maharashtra, India. Twenty-four participants (aged 30-60 years) with non-traumatic SCI and neurogenic bladder dysfunction were randomly allocated into two groups (n = 12 each). Group A (experimental) received PFN combined with conventional physiotherapy; group B (control) received conventional physiotherapy alone. Both protocols were delivered over two weeks. Outcome measures were the International Consultation on Incontinence Questionnaire (ICIQ) and the Spinal Cord Independence Measure (SCIM), assessed pre- and post-intervention using paired and independent samples t-tests.

Result

Group A showed a mean ICIQ reduction of 7.50 points (16.75 to 9.25; p < 0.001) and a mean SCIM gain of 21.08 points (46.25 to 67.33; p < 0.001). Group B showed a mean ICIQ reduction of 3.42 points (p < 0.01) and a SCIM gain of 10.25 points (p < 0.001). Between-group comparisons confirmed significantly superior outcomes in group A on both ICIQ (p < 0.01) and SCIM (p < 0.05), with improvements more than double those in the control group.

Conclusion

The addition of PFN to conventional physiotherapy significantly enhances bladder control and functional independence in non-traumatic post-operative SCI. These findings support the integration of neurodynamic techniques into standard SCI rehabilitation protocols.

## Introduction

Spinal cord injury (SCI) is among the most devastating neurological conditions, resulting in permanent or transient disruption of motor, sensory, and autonomic function below the level of injury [1]. The global burden is substantial, with an estimated 250,000-500,000 new cases occurring annually [2]. While traumatic aetiologies have historically predominated in the published literature, non-traumatic SCI, arising from degenerative myelopathy, spinal tumours, vascular insults, and inflammatory disorders, now accounts for 30-55% of all new SCI presentations in high-income countries, a proportion expected to rise further with ageing populations [3-5]. Non-traumatic SCI presents unique clinical challenges, including delayed diagnosis, atypical neurological presentations, and complex rehabilitation trajectories [6]. In the post-operative setting, following surgical decompression or spinal fixation, residual neurological deficits frequently persist, necessitating intensive and multidisciplinary rehabilitation [7].

Among the spectrum of residual impairments following SCI, neurogenic lower urinary tract dysfunction (NLUTD) affects more than 80% of individuals and is a primary determinant of long-term quality of life [8]. The neurological control of the lower urinary tract involves the pontine micturition centre, sacral spinal cord segments S2-S4, the pudendal nerve, and the autonomic nervous system [9]. Depending on injury level and completeness, NLUTD manifests as detrusor overactivity, detrusor underactivity, or detrusor sphincter dyssynergia, predisposing individuals to urinary tract infections, hydronephrosis, renal failure, and significant psychosocial burden [10-13].

The pelvic floor musculature plays a fundamental role in urinary continence through structural support of the bladder neck and neurally coordinated co-contraction with the external urethral sphincter [14]. In SCI, disruption of supraspinal control of pelvic floor motor neurons in Onuf's nucleus leads to impaired volitional activation and discoordination between detrusor and sphincter activity [15]. Pelvic floor muscle training (PFMT) has demonstrated efficacy across multiple populations; however, outcomes in SCI remain inconsistent, likely reflecting incomplete engagement of the neural pathways mediating pelvic floor control [16,17].

Neurodynamics, the application of mechanical and physiological principles to the nervous system, has emerged as an important adjunct in neurological physiotherapy [18]. Neural mobilisation techniques are proposed to reduce intraneural oedema, improve axoplasmic flow, decrease mechanosensitivity of sensitised neural tissue, and promote neural gliding [19]. The pudendal nerve (S2-S4), providing sensory and motor innervation to the pelvic floor and external urethral sphincter, and the femoral nerve (L2-L4), relevant in lumbar SCI populations, represent primary neurodynamic targets [20,21]. Pelvic floor neurodynamics (PFN) integrates targeted neurodynamic mobilisation of these structures with structured pelvic floor rehabilitation, potentially restoring axoplasmic transport, normalising afferent mechanosensitivity, and facilitating neuroplastic reorganisation within partially injured spinal cord segments [22]. Despite this biological plausibility, no randomised controlled trial (RCT) had previously evaluated PFN efficacy in non-traumatic post-operative SCI.

The present study was designed to evaluate the effects of a two-week PFN protocol on bladder control (International Consultation on Incontinence Questionnaire-Urinary Incontinence Short Form (ICIQ-UI SF)) and functional independence (Spinal Cord Independence Measure (SCIM)) in individuals with non-traumatic post-operative SCI compared with conventional physiotherapy alone.

## Materials and methods

Study design and participants

This prospective parallel-group RCT was conducted at Krishna College of Physiotherapy in Karad, Maharashtra, India, over a period of one year. Krishna Vishwa Vidyapeeth (Deemed to be University) Institutional Ethics Committee approval was obtained prior to commencement (protocol number: 280/2025-2026; approval number: KVV/IEC/10/2025), and all participants provided written informed consent. The trial was conducted in accordance with the Declaration of Helsinki and registered with the Clinical Trials Registry-India (CTRI) (registration number: CTRI/2026/06/111558).

Participants were recruited from the physiotherapy outpatient and inpatient departments. Inclusion criteria were as follows: male or female, aged 30-60 years; radiologically confirmed non-traumatic post-operative SCI (thoracic or lumbar level) based on magnetic resonance imaging (MRI) findings and diagnosis by a consultant neurosurgeon, with stable neurological status; presence of neurogenic bladder dysfunction (urgency, incontinence, or urinary retention); and willingness to participate. Exclusion criteria included the following: inability to follow verbal instructions; pelvic or urological surgery within the preceding six months; active pregnancy; and concurrent participation in another clinical trial. A total of 24 eligible participants were randomly allocated into two equal groups of 12 using a random number table sampling method.

Intervention

Both groups attended supervised sessions daily over a two-week period. Group A (experimental) received PFN combined with conventional physiotherapy; group B (control) received conventional physiotherapy alone. The detailed weekly intervention protocol for both groups is summarised in Table 1. The PFN protocol was structured in two progressive phases: week 1 focused on sensory awareness, pelvic floor activation, and non-provocative nerve gliding (femoral and pudendal nerve glides in supine), while week 2 advanced to coordination-based functional training, tension-based neurodynamic techniques, and bladder control in functional activities. This progression mirrors established neurodynamic principles of tissue-protective loading [19].

**Table 1:** Intervention PROM: passive range of motion; AAROM: active-assistive range of motion

Week	Group A (experimental group)	Group B (control group)
Week 1	Goal: awareness with sensory integration	Goal: maintain mobility
	Deep breathing exercises. Perineal stimulation (5 strokes × 3 reps). Femoral nerve glide in supine (10 reps × 3 sets). Pelvic floor awareness	Patient education. Deep breathing exercises. PROM exercises: lower limb (hip, knee, ankle; 10 × 2). Mat activities: rolling (5 times)
	Goal: activate the pelvic floor and improve nerve glide	Goal: enhance trunk and pelvic control for postural stability
	Glute squeezes: hold for 10 sec and then release (10 reps × 3 sets). Pelvic floor contraction: hold for 5-10 sec × 3 reps. Pudendal nerve glide with open knees (10 × 3). Pelvic floor contraction with the transverse abdominis: hold for 5-10 sec × 3 reps	Bed mobility. Trunk activation in hook lying (10 times). Sit on the edge of the bed with a supported trunk. Isometrics: quadriceps and hamstrings (10 sec hold × 3 sets)
	Goal: begin bladder control and improve detrusor control	Goal: improve functional independence
	Times voiding: try to urinate every 2-3 hours. Pelvic floor relaxation after voiding. Pelvic tilt (10 × 3). Pelvic floor contraction in sitting (10 sec hold × 3 reps)	Supported seated balance training with arms. Upper limb reachouts (10 times). Lower limb AAROM (10 × 2). Sit to stand with support (5 times)
Week 2	Goal: coordination training	Goal: enhance sitting tolerance and safe transfer
	Strengthen the pelvic floor and nerves together. Bridging with resistance band (10 sec hold × 5 reps). Ball squeeze (10 sec hold × 5 reps). Pudendal glide with pelvic tilting (10 glides × 3 sets). Seated postural correction + pelvic floor training	Dynamic trunk control (sitting; 10 times). Seated weight shifts with reachouts (10 times). Strengthening: quadriceps and hamstrings
	Goal: train bladder control in functional activities	Goal: standing and ambulation skills
	Pelvic floor with coughing (5 coughs with contraction × 2 sets). Half kneeling tilts (10 x 2). Sitting on a ball with contraction (10 sec hold × 3). Pudendal tension (modified slump; 3 times)	Supported standing (5 min). Weight shifts in standing. Marching in place with support (20 times). Ambulation with walker/assistant
	Goal: strengthen movement	Goal: functional task training
	Sit to stand with pelvic squeeze (10 sec × 3). Step-ups + pelvic floor contraction (10 sec × 3). Standing balance with contraction (5-10 sec × 3 reps)	Sit to stand without arm support (10 times). Functional activities. Ambulation inward
	Goal: reinforcement and re-evaluation; prepare for self-care	Goal: reinforcement and re-evaluation; prepare for self-care
	Patient education: diet and water. Pelvic floor rehabilitation exercises	Patient education: diet and water. Pelvic floor rehabilitation exercises

Outcome measures

Primary outcome measures were assessed at baseline (pre-intervention) and immediately following the completion of the two-week protocol (post-intervention).

The International Consultation on Incontinence Questionnaire-Urinary Incontinence Short Form (ICIQ-UI SF) is a validated, patient-reported outcome instrument assessing urinary incontinence symptoms and their impact on quality of life. Higher scores indicate greater symptom severity [18,19]. Written permission for use of the ICIQ-UI SF was obtained from the ICIQ Group, North Bristol NHS Trust, UK (June 2026).

The Spinal Cord Independence Measure (SCIM III) is a validated disability scale designed specifically for patients with spinal cord lesions, demonstrating superior responsiveness to rehabilitation-related change compared to the Functional Independence Measure [20,23,24].

Statistical analysis

Data were analysed using IBM SPSS Statistics for Windows, V. 25.0 (IBM Corp., Armonk, NY, USA). Normality was assessed using the Shapiro-Wilk test. Within-group pre-post comparisons used the paired samples t-test; between-group comparisons used the independent samples t-test. A p-value of <0.05 was considered statistically significant.

## Results

Demographic characteristics

Table 2 shows the age distribution of participants in both groups. The majority of participants belonged to the 50-59-year age group (n = 10; 42%). Group A showed equal distribution across all age groups, while group B had a higher proportion of participants in the 50-59-year category. Overall, the age distribution between groups was relatively comparable.

**Table 2:** Age distribution across both groups

Age group	Group A (experimental) (n = 12)	Group B (control) (n = 12)	Total
30-39 years	4 (33%)	3 (25%)	7 (29%)
40-49 years	4 (33%)	3 (25%)	7 (29%)
50-59 years	4 (33%)	6 (50%)	10 (42%)
Total	12 (100%)	12 (100%)	24 (100%)

The gender distribution of participants is detailed in Table 3. Females constituted the majority in both groups, accounting for seven participants (58.3%) in group A and nine participants (75%) in group B. Male participants represented five (41.7%) in group A and three (25%) in group B.

**Table 3:** Gender distribution

Gender	Group A (n = 12)	Group B (n = 12)
Female	7 (58.3%)	9 (75%)
Male	5 (41.7%)	3 (25%)
Total	12 (100%)	12 (100%)

Table 4 shows the distribution of spinal injury levels among participants in both groups. In the experimental group, thoracic injuries were more common (58.3%), whereas lumbar injuries predominated in the control group (58.3%).

**Table 4:** Level of spinal injury (affected region)

Level of injury	Group A (experimental)	Group B (control)
Thoracic	7 (58.3%)	5 (41.7%)
Lumbar	5 (41.7%)	7 (58.3%)
Total	12 (100%)	12 (100%)

Table 5 presents the within-group comparison of ICIQ and SCIM scores before and after the intervention. Group A demonstrated highly significant improvements in both outcome measures following the two-week PFN protocol. The mean ICIQ score decreased from 16.75 ± 1.96 to 9.25 ± 2.14 (mean change: -7.50 points; p < 0.001), while the mean SCIM score increased from 46.25 ± 7.13 to 67.33 ± 9.49 (mean change: +21.08 points; p < 0.001). Group B also showed significant improvements, with the mean ICIQ score decreasing from 16.42 ± 2.07 to 13.00 ± 2.49 (mean change: -3.42 points; p < 0.01) and the mean SCIM score increasing from 47.17 ± 7.46 to 57.42 ± 7.46 (mean change: +10.25 points; p < 0.001).

**Table 5:** Pre-post statistical summary: both groups ICIQ: International Consultation on Incontinence Questionnaire; SCIM: Spinal Cord Independence Measure

Group	Outcome	Pre-mean ± SD	Post-mean ± SD	Mean change	P-value
Group A	ICIQ	16.75 ± 1.96	9.25 ± 2.14	-7.50	<0.001
	SCIM	46.25 ± 7.13	67.33 ± 9.49	21.08	<0.001
Group B	ICIQ	16.42 ± 2.07	13.00 ± 2.49	-3.42	<0.01
	SCIM	47.17 ± 7.46	57.42 ± 7.46	10.25	<0.001

Table 6 demonstrates the superior effectiveness of the PFN intervention in group A compared to group B. Participants in the experimental group showed significantly greater improvement in urinary incontinence and functional independence, as evidenced by lower post-intervention ICIQ scores and higher SCIM scores. The magnitude of improvement in both outcomes was more than double in group A compared to the control group, indicating better clinical outcomes following the intervention.

**Table 6:** Between-group comparison of post-intervention scores and mean improvements ICIQ: International Consultation on Incontinence Questionnaire; SCIM: Spinal Cord Independence Measure

Outcome measure	Group A post-mean	Group B post-mean	Difference	P-value	
ICIQ score	9.25	13.00	-3.75	<0.01 (favours A)	
SCIM score	67.33	57.42	9.91	<0.05 (favours A)	

## Discussion

This RCT provides the first formal evidence that PFN combined with conventional physiotherapy produces significantly superior improvements in bladder control and functional independence compared to conventional physiotherapy alone in non-traumatic post-operative SCI. The ICIQ reduction of 7.50 points in group A substantially exceeds the published minimal clinically important difference (MCID) of approximately 2-3 points for the ICIQ-UI SF, confirming genuine clinical benefit [23]. The SCIM gain of 21.08 points over two weeks is equally noteworthy, reflecting meaningful gains in sphincter management and functional independence domains in which the SCIM has demonstrated superior responsiveness compared to the Functional Independence Measure [24,25].

The mechanisms underlying group A's superior outcomes are likely multifactorial. Neural mobilisation of the pudendal nerve reduces mechanosensitivity of sensitised neural tissue, improves intraneural microcirculation, and facilitates axoplasmic transport along efferent motor and afferent sensory fibres [18,19]. In post-operative non-traumatic SCI, where residual neural viability exists in incomplete injury presentations, restoration of optimal neural tissue mechanics may re-engage partially intact supraspinal and spinal bladder control pathways. The sacral micturition centre at S2-S4 relies on intact afferent signalling via the pudendal nerve; improved afferent conduction following neurodynamic mobilisation may restore more physiological reflex bladder responses [9]. The femoral nerve gliding component is additionally relevant for the lumbar SCI subgroup, augmenting pelvic floor motor unit recruitment beyond that achievable through voluntary exercise alone [21]. The progressive protocol structure, from non-provocative nerve gliding in week 1 to tension-based functional techniques in week 2, mirrors established neurodynamic principles of tissue-protective loading progression [19]. Perineal stimulation activates pudendal afferents and modulates detrusor overactivity through spinal inhibitory interneuron pathways [26], while pelvic floor co-activation with transverse abdominis optimises the functional continence synergy [27,28]. Timed voiding further contributes by recalibrating the central perception of bladder fullness through the cortical inhibition of the pontine micturition centre [29].

Group B's significant improvements on both measures affirm the value of conventional physiotherapy in this population, consistent with existing evidence for PFMT in neurogenic bladder dysfunction [16,17,30]. However, group A's improvements were more than double on both measures, indicating that conventional therapy alone does not fully exploit the available therapeutic potential. Comparison with the broader literature is instructive: studies of PFMT in multiple sclerosis-related bladder dysfunction report ICIQ improvements of 2-4 points [16], while trials of transcutaneous tibial nerve stimulation report ICIQ improvements of 3-5 points over 12-week protocols [31], suggesting that the two-week PFN protocol achieves comparable or superior short-term efficacy with greater accessibility and lower resource requirements. SCIM improvements in group A also compare favourably with published SCI rehabilitation data reporting gains of 10-15 points over four-to-eight-week admissions [32]. From a neuroplasticity perspective, the two-week window is consistent with evidence demonstrating rapid activity-dependent spinal cord plasticity in incomplete SCI, where repetitive task-specific sensorimotor training induces the reorganisation of spinal interneuronal circuits within days to weeks [33].

This study has several limitations. The relatively small sample size (n = 24) may limit the generalisability of the findings to the broader population of individuals with non-traumatic post-operative SCI. Furthermore, the two-week intervention period did not permit the assessment of the long-term sustainability of treatment effects. The absence of objective urodynamic assessment, including parameters such as post-void residual volume and detrusor pressure, limited the comprehensive evaluation of bladder function and physiological changes following the intervention. Participant blinding was not feasible because of the nature of the intervention. In addition, the unequal distribution of thoracic and lumbar injuries between groups may have influenced treatment responses. Future studies should recruit larger multicentre cohorts with longer follow-up periods and incorporate objective urodynamic parameters, including post-void residual volume and detrusor pressure, to provide a more comprehensive evaluation of bladder function and strengthen the robustness of the findings.

## Conclusions

The addition of PFN to conventional physiotherapy significantly enhances bladder control and functional independence in patients with non-traumatic post-operative SCI, with improvements more than double those achieved by conventional physiotherapy alone on both ICIQ-UI SF and SCIM. These findings support the clinical integration of PFN techniques into standard SCI rehabilitation protocols as a promising, cost-effective, and non-invasive intervention for neurogenic bladder dysfunction. Future research with larger sample sizes, multicentre designs, longer follow-up periods, and urodynamic evaluation is recommended to consolidate these findings and guide clinical implementation.
